# Tracking the evolution of cancer cell populations through the mathematical lens of phenotype-structured equations

**DOI:** 10.1186/s13062-016-0143-4

**Published:** 2016-08-23

**Authors:** Tommaso Lorenzi, Rebecca H. Chisholm, Jean Clairambault

**Affiliations:** 1 School of Mathematics and Statistics, University of St Andrews, North Haugh, St Andrews, KY16 9SS UK; 2 School of Biotechnology and Biomolecular Sciences, University of New South Wales, NSW, Sydney, 2052 Australia; 3 INRIA Paris Research Centre, MAMBA team, 2, rue Simone Iff, CS 42112, Paris Cedex 12, 75589 France; 4 Sorbonne Universités, UPMC Univ. Paris 6, UMR 7598, Laboratoire Jacques-Louis Lions, Boîte courrier 187, 4 Place Jussieu, Paris Cedex 05, 75252 France

**Keywords:** Cancer cell populations, Phenotypic evolution, Natural selection, Phenotype plasticity, Epimutations, Cytotoxic-drug resistance, Phenotypic heterogeneity, Mathematical oncology, Phenotype-structured equations

## Abstract

**Background:**

A thorough understanding of the ecological and evolutionary mechanisms that drive the phenotypic evolution of neoplastic cells is a timely and key challenge for the cancer research community. In this respect, mathematical modelling can complement experimental cancer research by offering alternative means of understanding the results of in vitro and in vivo experiments, and by allowing for a quick and easy exploration of a variety of biological scenarios through *in silico* studies.

**Results:**

To elucidate the roles of phenotypic plasticity and selection pressures in tumour relapse, we present here a phenotype-structured model of evolutionary dynamics in a cancer cell population which is exposed to the action of a cytotoxic drug. The analytical tractability of our model allows us to investigate how the phenotype distribution, the level of phenotypic heterogeneity, and the size of the cell population are shaped by the strength of natural selection, the rate of random epimutations, the intensity of the competition for limited resources between cells, and the drug dose in use.

**Conclusions:**

Our analytical results clarify the conditions for the successful adaptation of cancer cells faced with environmental changes. Furthermore, the results of our analyses demonstrate that the same cell population exposed to different concentrations of the same cytotoxic drug can take different evolutionary trajectories, which culminate in the selection of phenotypic variants characterised by different levels of drug tolerance. This suggests that the response of cancer cells to cytotoxic agents is more complex than a simple binary outcome, *i.e.*, extinction of sensitive cells and selection of highly resistant cells. Also, our mathematical results formalise the idea that the use of cytotoxic agents at high doses can act as a double-edged sword by promoting the outgrowth of drug resistant cellular clones. Overall, our theoretical work offers a formal basis for the development of anti-cancer therapeutic protocols that go beyond the ‘maximum-tolerated-dose paradigm’, as they may be more effective than traditional protocols at keeping the size of cancer cell populations under control while avoiding the expansion of drug tolerant clones.

**Reviewers:**

This article was reviewed by Angela Pisco, Sébastien Benzekry and Heiko Enderling.

**Electronic supplementary material:**

The online version of this article (doi:10.1186/s13062-016-0143-4) contains supplementary material, which is available to authorized users.

## Background

A growing body of evidence indicates that cancer progression at the cellular level is, in essence, an evolutionary process [1–6]. During cancer progression, novel phenotypic variants emerge via heritable changes in gene expression. Subsequently, phenotypic variants are subject to natural selection – they survive, reproduce, and die – under the action of the tumour microenvironment and anti-cancer agents. The scenario is further complicated by cell-to-cell variability in gene expression, which gives rise to phenotypic differences between cancer cells of the same population. This phenotypic heterogeneity is a dynamic source of therapeutic resistance that needs to be accounted for when investigating effective anti-cancer therapeutic protocols [7].

Novel phenotypic variants in cancer cell populations originate mainly from mutations (*i.e.*, genetic modifications). However, novel phenotypic variants can also emerge due to epimutations (*i.e.*, heritable changes in gene expression that leave the sequence of bases in the DNA unaltered) [8–12]. For instance, recent experiments using fluorescent-activated cell sorting have demonstrated that non-genetic instability mediated by fluctuating protein levels allows cancer cells to reversibly transition between different phenotypic states [13–15]. Such non-genetic source of phenotypic variability has been increasingly recognised as integral to the development of resistance to cytotoxic agents in cancer cell populations [16, 17].

Identifying the ecological and evolutionary mechanisms that drive the phenotypic evolution of neoplastic cell populations is, therefore, a timely and key challenge for the cancer research community. In this regard, mathematical modelling provides additional tools in the fight against cancer, and many biologists and clinicians are now looking for a mathematical approach to complement their research [18–27]. Among others, models arising from mathematical ecology enable falsification of biological hypotheses concerning the principles that underpin the dynamics of neoplastic cell populations, and extrapolation beyond scenarios analysed by means of in vitro and in vivo experiments [28].

During the last fifty years, partial differential equations (PDEs) for populations structured by physiological traits have been extensively used to achieve a better understanding of a wide range of ecological phenomena [29, 30]. These equations describe population dynamics in terms of the evolution of population densities across phenotypic spaces, and can be derived from individual-based (IB) models through suitable asymptotic limits [31–33]. However, unlike IB models, which can be explored mainly through numerical simulations only, PDEs for populations structured by physiological traits make it possible to integrate numerical simulations with rigorous analysis, in order to achieve more robust biological conclusions [34].

In particular, we have recently shown that phenotype-structured PDEs constitute a convenient apparatus to study *in silico* the dynamics of cancer cell populations. In more detail, we formulated a PDE model for the coevolution of a population of healthy cells and a population of cancer cells structured by the level of resistance to a cytotoxic drug [35]. Further, we extended this model to consider cell populations structured also by a spatial variable [36]. Most recently, we presented a PDE model of phenotypic evolution in a cancer cell population structured by the expression levels of two phenotypic traits, survival potential and proliferation potential [37]. Overall, the results of our analyses and numerical simulations provide a new perspective on the inherent risks of interventional chemotherapy in cancer patients by showing how the adaption of even nongenetically unstable cell populations exposed to antiproliferative drugs can be acted upon by selective forces, which drive the outgrowth of drug resistant cell clones.

To investigate the roles of phenotype plasticity and selection pressures in tumour relapse, here we propose a phenotype-structured PDE model of evolutionary dynamics in a cancer cell population which is exposed to the action of a cytotoxic drug within an in vitro culture system. Our model is informed by a previous conceptual model [38] and focuses on a cancer cell population structured by the expression level of a gene which is linked to both the cellular levels of cytotoxic-drug resistance and proliferative potential – such as ALDH1, CD44, CD117 or MDR1 [39, 40]. We characterise the phenotypic state of each cell by means of a continuous variable related to the level of expression of this gene, and we allow the cell phenotypic state to change in time due to non-genetic instability, which is mediated by random epimutation events. The inclusion of a dynamic continuous population structure and its plasticity makes PDE models a natural framework to study, *in silico*, the phenotypic evolution of cancer cells. Note that, in the model presented here, non-genetic instability is purely random and represented by a constant rate multiplying a diffusion operator. In more sophisticated models involving metabolism-dependent epigenetic dynamics, as those advocated in [41], this constant would be replaced by a suitable function to incorporate the effects of the cell load in histone demethylases, DNA methyltransferases, or other epigenetic enzymes, together with their substrates and coenzymes.

Exploiting the analytical tractability of our model, we dissect the relative contributions of random epimutations, natural selection and competition for resources as drivers of adaptation in cancer cell populations that evolve under the action of cytotoxic drugs. This analytical work builds on recent advances in the study of non-local PDEs that arise in models of population dynamics [33, 42, 43], which enable us to explore the parameter space of our model completely. To illustrate analytical results through numerical simulations, we calibrate our model by means of existing data from in vitro experiments on the phenotypic evolution of HL60 leukemic cells exposed to vincristine [14]. Nonetheless, the results of our analyses are qualitatively robust across a wide range of parameter values. This makes the conclusions presented in our current work applicable to a broad spectrum of cancer cell lines evolving under the action of different cytotoxic agents.

Our main findings elucidate how the phenotype distribution, the level of phenotypic heterogeneity and the size of cancer cell populations exposed to cytotoxic drugs are shaped by the strength of natural selection, the rate of random epimutations, the intensity of the competition for limited resources between cells, and the drug dose in use. Moreover, we are able to obtain exact conditions for the successful adaptation of cancer cells, and we find that whilst phenotypic plasticity may facilitate adaptation in neoplastic cell populations exposed to cytotoxic drugs, frequent fluctuations in protein levels can also contribute to extinction in the presence of high drug concentrations. Finally, our mathematical results provide a ‘proof of concept’ of the hypothesis that the use of cytotoxic agents at high doses is a double-edged sword in the fight against cancer, and offer a formal basis for the development of therapeutic protocols which effectively exploit evolutionary mechanisms to improve the efficacy of cytotoxic therapy.

## Methods

We study evolutionary dynamics in a well-mixed population of cancer cells that is structured by the expression level $y \in \mathbb {R}_{+}$ of a gene which is linked to both the cellular levels of cytotoxic-drug resistance and proliferative potential. Following Pisco and Huang [38], we assume that there is a level of expression *y*^*H*^ which endows cells with the highest level of cytotoxic-drug resistance, and a level of expression *y*^*L*^<*y*^*H*^ conferring the highest proliferative potential when there are no xenobiotic agents. In this framework, we characterise the phenotypic state of each cell by means of the variable $x \in \mathbb {R}$ with

$$x = \frac{y-y^{L}}{y^{H}-y^{L}}, $$

so that the state *x*=1 corresponds to the highest level of cytotoxic-drug resistance, while the state *x*=0 corresponds to the highest level of proliferative potential in the absence of xenobiotic agents.

Cells inside the population proliferate or die, compete for limited resources, and undergo variation in phenotype due to random epimutation events. Furthermore, a cytotoxic drug can be present, which acts by increasing the death rate of cancer cells. The function *n*(*x*,*t*)≥0 stands for the population density, so that the total number of cells at time *t* is computed as

1 $$ \rho(t) = \int_{\mathbb{R}} n(x,t) \; \mathrm{d}x,  $$

while the average phenotypic state and the related variance at time *t* are computed, respectively, as

2 $$ \begin{aligned} \mu(t) &= \frac{1}{\rho(t)} \int_{\mathbb{R}} x \; n(x,t) \; \mathrm{d}x,\\ \sigma^{2}(t) &= \frac{1}{\rho(t)} \int_{\mathbb{R}} x^{2} \; n(x,t) \; \mathrm{d}x - \mu(t)^{2}. \end{aligned}  $$

In this mathematical framework, the function *σ*^2^(*t*) provides a measure of the level of intrapopulation heterogeneity at time *t*. Finally, we introduce the function *c*(*t*)≥0 to model the concentration of cytotoxic drug at time *t*. Throughout the paper, we will mainly focus on the case of continuous drug infusion, and we will use the parameter *C*>0 to model the constant concentration of cytotoxic drug inside the system.

### PDE model

We describe the evolution of the cell population density *n*(*x*,*t*) by means of the following phenotype-structured PDE

3 $$ \frac{\partial n}{\partial t} (x,t) = \underbrace{\beta \; \frac{\partial^{2} n}{\partial x^{2}}(x,t)}_{\substack{\text{\scriptsize{non-genetic }}\\\text{\scriptsize{instability}}}} + \underbrace{R\left(x,\rho(t),c(t)\right) n(x,t)}_{\text{\scriptsize{natural selection}}},  $$

which we complete with the boundary conditions

4 $${} n(x,\cdot) \rightarrow 0 ~~ \text{and}~~~\frac{\partial^{q} n}{\partial x^{q}}(x,\cdot) \rightarrow 0~\text{for all}\, q\in\mathbb{N}~~\text{~as}~|x|\rightarrow \infty  $$

and the initial condition

5 $$ n(x,0) \in L^{1} \cap L^{\infty}(\mathbb{R}), \quad n(x,0) > 0~ \text{a.e. on}~\Omega \subset \mathbb{R}.  $$

In the above equation, *Ω* is a compact subset of $\mathbb {R}$. Eq. (3) relies on the assumptions and the modelling strategies presented in the following subsections.

#### Mathematical modelling of non-genetic instability

To reduce biological complexity to its essence, we make the *prima facie* assumption that random epimutations yield infinitesimally small phenotypic modifications [44, 45]. Therefore, we model the effects of non-genetic instability through a diffusion operator. The diffusion coefficient *β*>0 in Eq. (3) stands for the rate of epimutation of cancer cells, which is assumed to be constant, as mentioned earlier.

#### Mathematical modelling of natural selection

Natural selection is driven here by the function *R*(*x*,*ρ*(*t*),*c*(*t*)), which represents the fitness of cancer cells in the phenotypic state *x* at the time *t*, given the total number of cells *ρ*(*t*) and the concentration of cytotoxic drug *c*(*t*). Throughout this paper, we make use of the following definition:

6 $$ R\left(x,\rho(t),c(t)\right) := p(x) - d \rho(t) - k(x,c(t)).  $$

Definition (6) relies on the idea that a higher total number of cells corresponds to less available resources; therefore, we let cells inside the population die at rate *dρ*(*t*), where the parameter *d*>0 models the rate of death due to intrapopulation competition. The function *p*(*x*) stands for the net proliferation rate of cancer cells in the phenotypic state *x*, while the function *k*(*x*,*c*(*t*)) is the rate of death caused by the cytotoxic drug. Since the phenotypic state *x*=1 corresponds to the highest level of cytotoxic-drug resistance, we assume that the function *k* is strictly convex with minimum in *x*=1. Furthermore, because the death rate of cancer cells will increase as the concentration of the cytotoxic drug increases, we assume that *k* is an increasing function of *c*. On the other hand, to take into account the fact that the phenotypic state *x*=0 corresponds to the highest level of proliferative potential when there are no xenobiotic agents (*i.e.*, when *c*(·)=0), we assume that *p* is a strictly concave function with maximum in *x*=0. In this setting, we follow the modelling strategies presented in [42] and define the functions *p* and *k* as:

7 $$ p(x) := \gamma - \eta \; x^{2}, \qquad \kappa(x,c(t)) := c(t) \; (x-1)^{2}.  $$

In the above definitions, the parameter *γ*>0 corresponds to the maximum fitness of cancer cells, and the non-linear selection gradient *η*>0 provides a measure of the strength of natural selection in the absence of xenobiotic agents.

### Setup of numerical simulations and model parametrisation

We numerically solve the mathematical problem defined by completing Eq. (3) with boundary conditions (4) and the following initial condition



where *b* and *L* are positive numbers, *a*_*i*_>0 are uniformly distributed random numbers between 0 and 1, *x*_*i*_ are uniformly distributed random numbers between −*L* and *L*, and *K*>0 is the carrying capacity of the population. The above definition represents an initial population composed of different cellular clones labelled by the index *i*=1,…,*N*. To perform numerical simulations, we set *N*=20.

Numerical computations are performed in MATLAB. We select a uniform discretisation consisting of 1200 points on the interval [−2*L*,2*L*] with *L*=2 as the computational domain. The method for calculating numerical solutions is based on a time splitting scheme between the conservative part and the reaction term [46]. As for the conservative part, we approximate the diffusion term through a three-point explicit scheme. On the other hand, we use an implicit-explicit finite difference scheme for the reaction term [35]. For all simulations, we set the time step *dt*=10^−4^, in order to ensure the stability of the numerical scheme.

We set the maximum fitness *γ*=0.66, so that the doubling time of cells in the highly proliferative state *x*=0 is about 25 h (*cf.* data in [47]). Furthermore, the in vitro experiments presented in [14] on the phenotypic evolution of HL60 leukemic cells exposed to vincristine have shown that, in the absence of xenobiotic agents, highly cytotoxic-drug resistant cells take approximatively 18 days to accomplish the repopulation of the equilibrium cell distribution observed without xenobiotic agents. Also, according to the same experiments, the ratio between the proliferation rate of the cells with the highest level of cytotoxic-drug resistance and the proliferation rate of the cells with the highest proliferative potential is equal to 5. Therefore, we choose the non-linear selection gradient *η* and the rate of epimutations *β* to be such that, when *c*(·)=0, it takes approximatively 18 days for an initial population mainly composed of cells in the phenotypic state *x*=1 to reconstitute the equilibrium phenotypic distribution corresponding to *c*(·)=0, with the value of *η* being constrained by the condition *p*(*x*=0)/*p*(*x*=1)=5 (*vid.* Additional file 1). Moreover, we define the average rate of death due to intrapopulation competition as *d*:=*γ*/*K*, so that the equilibrium value of the total number of cells in the absence of xenobiotic agents and without epimutations (*i.e.*, when *c*(·)=0 and *β*=0) is equal to the carrying capacity *K*=10^8^ (*cf.* Figure S5 in [14]). Based on these considerations, unless otherwise stated, we perform numerical simulations using the parameter values listed in Table 1, which are consistent with previous reports [14, 47–49]. Finally, the concentration of cytotoxic drug is expressed in terms of the LC_*α*_ – *i.e.*, the value of *c* that is required to reduce the equilibrium value of the total number of cells by *α*%.

**Table 1:** Values of the parameters used to perform numerical simulations

Parameter	Biological meaning	Value
*γ*	Maximum fitness	0.66 per day
*η*	Selection gradient	0.132 per day
*d*	Rate of death due to intrapopulation competition	0.66×10^−8^ per day
*β*	Rate of epimutation	0.01 per day

## Results and discussion

### Cell dynamics in the absence of xenobiotic agents

As mentioned earlier, in the framework of our model, the total number of cells *ρ*(*t*), the average phenotypic state *μ*(*t*) and the level of intrapopulation heterogeneity *σ*^2^(*t*) are computed according to Eqs. (1)–(2). Then, in the absence of xenobiotic agents (*i.e.*, when *c*(·)=0), a complete characterisation of the cancer cell population at equilibrium is provided by the following theorem:

#### **Theorem 1**

Let *c*(·)=0, and denote by $\overline {n}(x)$ the equilibrium population density for *c*(·)=0. Then:

(i) if *γ*−(*βη*)^1/2^≤0,
  9 $$ \rho(t) \rightarrow 0, \qquad \text{as }\, t \rightarrow \infty;  $$
(ii) if *γ*−(*βη*)^1/2^>0,
  10 $$ \rho(t) \rightarrow \overline{\rho} = \frac{1}{d}\left[ \gamma-(\beta\eta)^{1/2} \right], \qquad \text{as }\, t \rightarrow \infty  $$
  and
  11 $$ \overline{n}(x) = \overline{\rho} \; \frac{{(\eta/\beta)^{1/4}}}{(2\pi)^{1/2}} \exp\left[-\frac{1}{2}\left(\frac{\eta}{\beta}\right)^{1/2} x^{2}\right],  $$
  so that
  12 $$ \mu(t) \rightarrow \overline{\mu} = 0~~\text{and} \quad \sigma^{2}(t) \rightarrow \overline{\sigma}^{2} = \sqrt{\frac{\beta}{\eta}},~~~\text{as }\, t \rightarrow \infty.  $$

The results established by Theorem 1 are discussed, as points R1 - R4 below, and illustrated by means of numerical simulations in the following subsections. The proof is detailed in Appendix Appendix 1. Proofs of Theorem 1 and Theorem 2 – to which we refer the mathematically inclined reader – and it follows from a general analysis that we have presented in two recent papers [33, 42].

#### R1. Higher rates of random epimutations can drive the cancer cell population to extinction under strong selective pressures

Point (i) of Theorem 1 shows that the total number of cells *ρ*(*t*) converges to zero when the value of the product between the epimutation rate *β* and the non-linear selection gradient *η* exceeds the square of the maximum fitness *γ*. Hence, in the presence of large selection gradients, high rates of random epimutations increase the probability of ultimate extinction. This suggests that frequent random epimutations can drive cancer cell populations to extinction under strong selective pressures.

#### R2. The population evolves to be mainly composed of highly proliferative cells

Point (ii) of Theorem 1 demonstrates that, for any initial population density *n*(*x*,0), the equilibrium phenotype distribution $\overline {n}(x)$ is unimodal with the average phenotypic state $\overline {\mu }$ being at the maximum point of the distribution *x*=0, and the total number of cells converges to the stable positive value $\overline {\rho }$. This holds true for all parameter settings, on the condition that the maximum fitness *γ*, the non-linear selection gradient *η* and the rate of random epimutations *β* satisfy the relation *γ*−(*βη*)^1/2^>0.

**Fig. 1:**
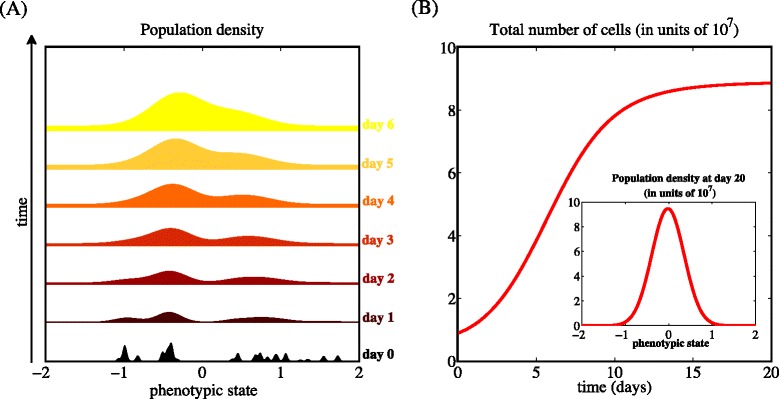
In the absence of drug, the population evolves to be mainly composed of highly proliferative cells. Sample dynamics of the population density *n*(*x*,*t*) (panel **a**) and the total number of cells *ρ*(*t*) (panel **b**) in the absence of xenobiotic agents (*i.e.*, when *c*(·)=0), for the parameter values listed in Table 1. The initial condition corresponds to a population composed of different cellular clones [*cf.* Eq. (??)]. The inset of panel (**b**) displays the population density after 20 days [*i.e.*, *n*(*x*,20)], which coincides with the equilibrium population density $\overline {n}(x)$ of Eq. (11)

These results are illustrated by the plots in Fig. 1, which present sample dynamics of the population density *n*(*x*,*t*) and the total number of cells *ρ*(*t*) when *c*(·)=0, for the parameter values listed in Table 1. Independently of the phenotypic composition of the initial population, cells in the phenotypic state *x*=0 (*i.e.*, the phenotypic state corresponding to the highest level of proliferative potential in the absence of xenobiotic agents) become the most abundant within the population [*vid.* Fig. 1a and inset of Fig. 1b], and the total number of cells reaches the stable value $\overline {\rho }$ [*vid.* Fig. 1b].

#### R3. The number of cells and the level of intrapopulation heterogeneity at equilibrium depend on the rate of random epimutations and on the strength of natural selection

**Fig. 2:**
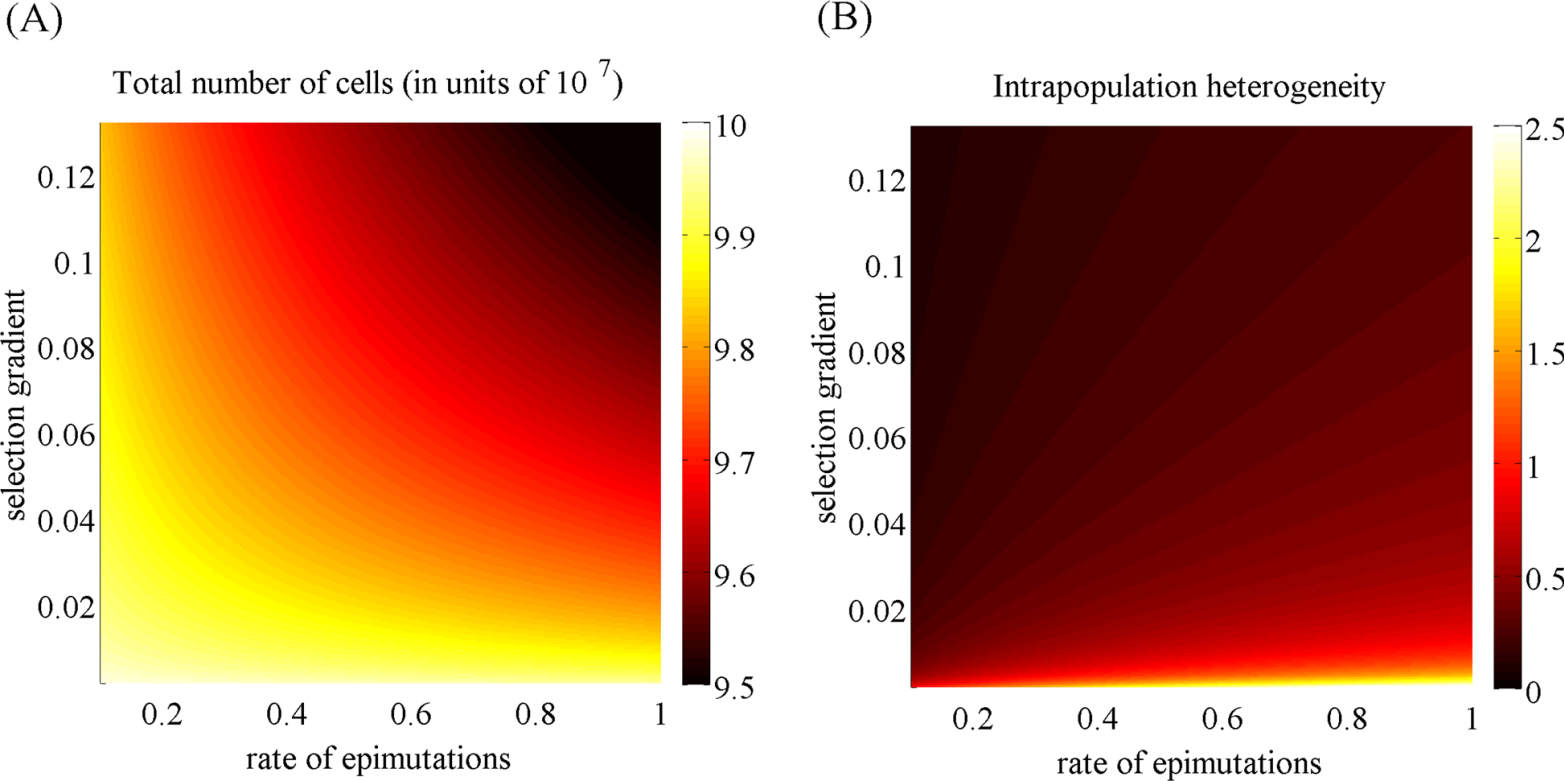
In the absence of drug, the number of cells and the level of intrapopulation heterogeneity at equilibrium depend on the rate of random epimutations and on the strength of natural selection. Plot of the total number of cells $\overline {\rho }$ (panel **a**) and the level of intrapopulation heterogeneity $\overline {\sigma }^{2}$ (panel **b**) associated with the equilibrium phenotypic distribution as a function of the epimutation rate *β* (in units of 10^−2^) and the selection gradient *η*. The values of the other model parameters are as in Table 1

Point (ii) of Theorem 1 also sheds some light on the way the size of the population and the level of phenotypic diversity are affected by non-genetic instability and natural selection. This is highlighted by the phase diagrams in Fig. 2, which show how the total number of cells $\overline {\rho }$ and the level of intrapopulation heterogeneity $\overline {\sigma }^{2}$ at equilibrium vary as a function of the rate of epimutations *β* and the non-linear selection gradient *η*. In summary, the total number of cells is a decreasing function of *β* and *η*, while the level of intrapopulation heterogeneity increases with *β* and decreases with *η*. These results formalise the idea that more intense non-genetic instability and stronger selection pressures bring about cancer cell populations of a smaller size. Also, more frequent phenotypic fluctuations lead to higher levels of intrapopulation heterogeneity, whilst intense selective pressures support less phenotypic diversity.

#### R4. The population size at equilibrium depends also on the maximum fitness and on the rate of cell death due to intrapopulation competition

**Fig. 3:**
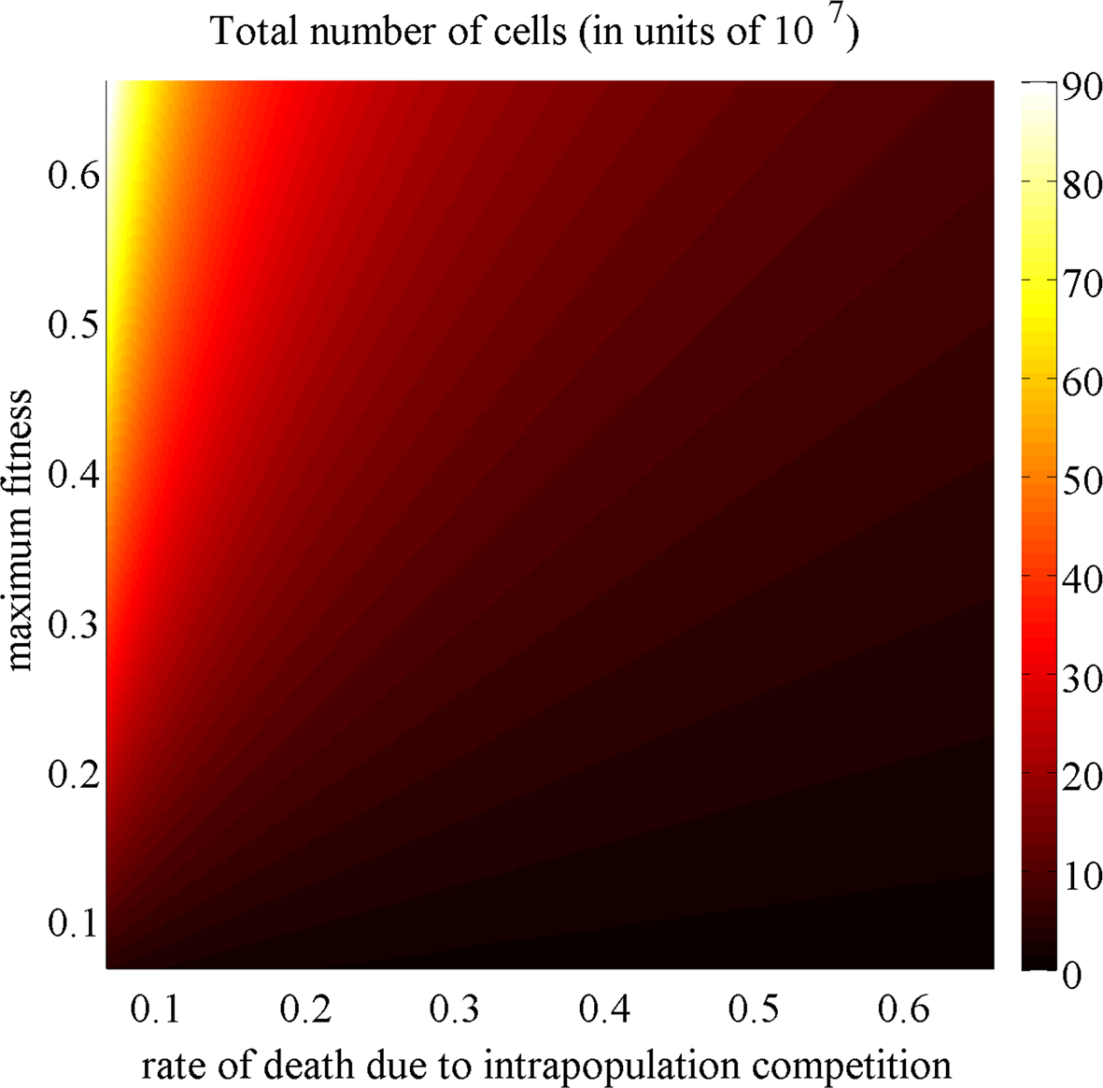
In the absence of drug, the population size at equilibrium depends also on the maximum fitness and on the rate of cell death due to intrapopulation competition. Plot of the total number of cells at equilibrium $\overline {\rho }$ as a function of the rate of death due to intrapopulation competition *d* (in units of 10^−8^) and the maximum fitness *γ*. The values of the other model parameters are as in Table 1

The results established by point (ii) of Theorem 1 and illustrated by the phase diagram in Fig. 3 indicate, as one could expect, that the total number of cells at equilibrium is an increasing function of the maximum fitness *γ* and a decreasing function of the average rate of death due to intrapopulation competition *d*. In the framework of our model, lower values of the parameter *γ* are associated with more hostile environmental conditions, while higher values of the parameter *d* correspond to stronger intrapopulation competition. Therefore, these results formalise the idea that cancer cell populations shrink in the presence of harsh environments and intense cellular competition for limited resources.

### Cell dynamics under the action of cytotoxic drug

**Fig. 4:**
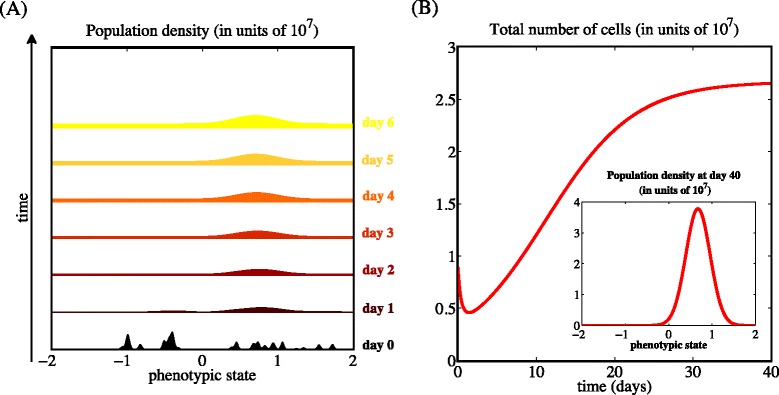
The cytotoxic drug triggers a population bottleneck. Sample dynamics of the population density *n*(*x*,*t*) (panel **a**) and the total number of cells *ρ*(*t*) (panel **b**) in the case when the drug concentration *c*(·)=*C* corresponds to the LC_70_ dose. The values of the model parameters are as in Table 1, and the initial condition corresponds to a population composed of different cellular clones [*cf.* Eq. (??)]. The inset of panel (**b**) displays the population density after 40 days [*i.e.*, *n*(*x*,40)], which coincides with the equilibrium population density $\overline {n}_{c}(x)$ of Eq. (16)

The plots in Fig. 4 depict sample dynamics of the population density *n*(*x*,*t*) and the total number of cells *ρ*(*t*) in the case when the drug concentration *c*(·)=*C* corresponds to the LC_70_ dose, for the parameter values listed in Table 1. These plots illustrate how the cytotoxic drug triggers a population bottleneck by causing a sharp reduction in the number of cells, followed by approximatively 1 day of relatively constant population level before cells in a phenotypic state close to *x*=1 (*i.e.*, the phenotypic state corresponding to the highest level of cytotoxic-drug resistance) are selected and start proliferating. Subsequently, the population size grows again and, ultimately, attains a stable value.

Although the phenotype distribution and the number of cells at equilibrium, as well as the time required for the population to recover, vary depending on the choice of the drug dose, the dynamics of the population density and the total number of cells remain qualitatively similar to those shown in Fig. 4. A complete characterisation of the cancer cell population at equilibrium is provided by the following theorem:

#### **Theorem 2**

Let *c*(·)=*C*>0, define

13 $$ \gamma_{c} := \gamma - \frac{\eta \; C}{\eta + C}, \qquad \eta_{c} := \eta + C,  $$

and denote by $\overline {n}_{c}(x)$ the equilibrium population density for *c*(·)=*C*. Then:

(i) if *γ*_*c*_−(*βη*_*c*_)^1/2^≤0,
  14 $$ \rho(t) \rightarrow 0, \qquad \text{as }\, t \rightarrow \infty;  $$
(ii) if *γ*_*c*_−(*βη*_*c*_)^1/2^>0,
  15 $$ \rho(t) \rightarrow \overline{\rho}_{c} = \frac{1}{d}\left[\gamma_{c}-(\beta\eta_{c})^{1/2} \right], \qquad \text{as }\, t \rightarrow \infty  $$
  and
  16 $$ \overline{n}_{c}(x) = \overline{\rho}_{c} \; \frac{{(\eta_{c}/\beta)^{1/4}}}{(2\pi)^{1/2}} \exp\left\{-\frac{1}{2}\left(\frac{\eta_{c}}{\beta}\right)^{1/2} \left[x-\overline{x}_{c}\right]^{2}\right\},  $$
  with
  17 $$ \overline{x}_{c} := \frac{C}{\eta+C},  $$
  so that
  18 $${} \mu(t) \rightarrow \overline{\mu}_{c} = \overline{x}_{c}~~\text{and} \quad \sigma^{2}(t) \rightarrow \overline{\sigma}^{2}_{c} = \sqrt{\frac{\beta}{\eta_{c}}},~~\text{as }\, t \rightarrow \infty.  $$

As explained in Appendix Appendix 2: Proof of Theorem 2, the proof of Theorem 2 follows the method of proof of Theorem 1. Here we proceed by discussing, as points R5 - R8 below, the pieces of biological information which are conveyed by the above mathematical results. Furthermore, in order to extend the analytical results established by Theorem 2, we perform numerical simulations letting the drug concentration vary over time. The obtained results are discussed as points R9 and R10 below.

#### R5. Higher rates of random epimutations can drive the population to extinction in the presence of high drug doses

Point (i) of Theorem 2 shows that the total number of cells *ρ*(*t*) converges to zero when the value of the product between the rate of random epimutations *β* and the sum of the non-linear selection gradient *η* and the drug concentration *C* is larger than the square of the maximum fitness *γ*. This supports the idea that higher rates of random epimutations increase the chance that cancer cell populations go extinct when exposed to sufficiently high concentrations of cytotoxic agents.

#### R6. Higher drug doses correspond to lower cell numbers

**Fig. 5:**
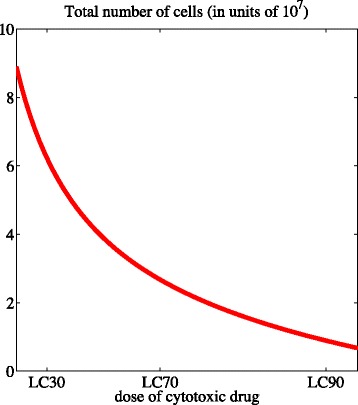
Higher drug doses correspond to lower cell numbers. Plot of the total number of cells at equilibrium $\overline {\rho }_{c}$ as a function of the drug concentration *c*(·)=*C* for the parameter values listed in Table 1

Point (ii) of Theorem 2 reveals that, if the condition *γ*_*c*_−(*βη*_*c*_)^1/2^>0 is satisfied, the total number of cells *ρ*(*t*) converges to the stable positive value $\overline {\rho }_{c}$. By analogy with the case when there is no cytotoxic drug inside the system, $\overline {\rho }_{c}$ increases with the maximum fitness *γ*, and decreases with the non-linear selection gradient *η*, the rate of epimutations *β*, and the rate of cell death due to intrapopulation competition *d*. Moreover, as one could expect, the cytotoxic drug has a detrimental effect on the population size, and the number of cells at equilibrium is a decreasing function of the drug dose *C* [*vid.* Fig. 5].

#### R7. Higher drug doses promote a selective sweep towards more resistant phenotypic variants

**Fig. 6:**
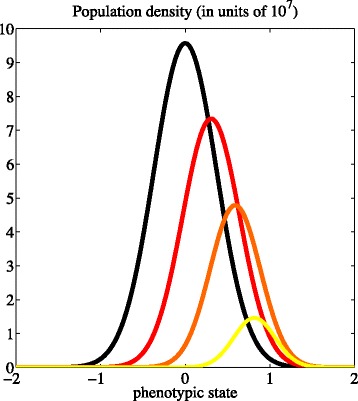
Higher drug doses promote a selective sweep towards more resistant phenotypic variants. Profile of the equilibrium population density $\overline {n}_{c}(x)$ for different values of the drug dose, *i.e.*, *c*(·)=0 (*black line*), *c*(·)=*C*≡ LC_30_ (*red line*), *c*(·)=*C*≡ LC_60_ (*orange line*), and *c*(·)=*C*≡ LC_90_ (*yellow line*). The values of the model parameters are as in Table 1

Point (ii) of Theorem 2 demonstrates that – for all values of the model parameters satisfying the condition *γ*_*c*_>(*βη*_*c*_)^1/2^ – the equilibrium phenotype distribution given by $\overline {n}_{c}(x)$ is unimodal with the average phenotypic state $\overline {\mu }_{c}$ being at the maximum point of the distribution $\overline {x}_{c}$. Also, the population density at equilibrium does not depend on the initial population density. As illustrated by Fig. 6, the values of $\overline {x}_{c}$ and $\overline {\mu }_{c}$ continuously increase from 0 to 1 as the drug dose increases.

The results established by Point (ii) of Theorem 2 indicate that, during cytotoxic-drug treatment, the surviving cells can reside within a spectrum of intermediate phenotypic states – ranging from less drug resistant states to more drug tolerant states – depending on the dose in use. These results indicate that different concentrations of the same cytotoxic drug can lead to the selection of cancer cells in different phenotypic states. In particular, while high doses confer a competitive advantage to highly resistant phenotypic variants, lower concentrations of cytotoxic drugs promote the selection of intermediate levels of cytotoxic-drug resistance and proliferative potential, which may result in an advantageous therapeutic outcome.

#### R8. The level of intrapopulation heterogeneity decreases as the drug dose increases

**Fig. 7:**
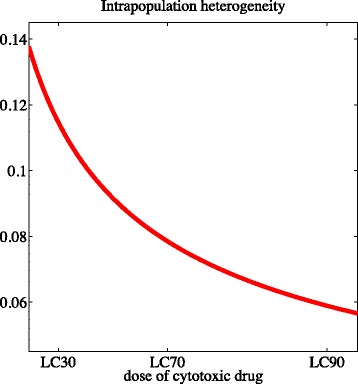
The level of intrapopulation heterogeneity decreases as the drug concentration increases. Plot of the level of intrapopulation heterogeneity at equilibrium $\overline {\sigma }^{2}_{c}$ as a function of the drug concentration *c*(·)=*C* for the parameter values listed in Table 1

As in the case when there is no cytotoxic drug, the level of intrapopulation heterogeneity at equilibrium $\overline {\sigma }^{2}_{c}$ is an increasing function of the rate of random epimutations *β*, and a decreasing function of the non-linear selection gradient *η*. Furthermore, as illustrated by Fig. 7, point (ii) of Theorem 2 reveals that $\overline {\sigma }^{2}_{c}$ is a decreasing function of the drug concentration *C*.

#### R9. The cancer cell population regains cytotoxic-drug sensitivity when drug delivery is discontinued

In order to extend the analytical results established by Theorem 2, we perform numerical simulations letting the drug concentration vary over time [*i.e.*, we consider the case in which the drug concentration is modelled by a function of time *c*(*t*)]. In particular, we explore the dynamics of the cancer cell population exposed to a piecewise-continuous periodic delivery of the cytotoxic drug. As illustrated by the results presented in the Additional file 2, our model predicts the selection of cells in a more resistant phenotypic state in the presence of the cytotoxic drug, whereas selection is reverted in favour of drug-sensitive cells when drug delivery is discontinued. Also, the same results clearly show that administering the cytotoxic drug in an alternate fashion leads to the emergence of cycling subpopulations of drug-resistant and drug-sensitive cells.

#### R10. Comparing the administration of high-drug doses separated by drug-free periods with the continuous delivery of relatively low-drug doses

**Fig. 8:**
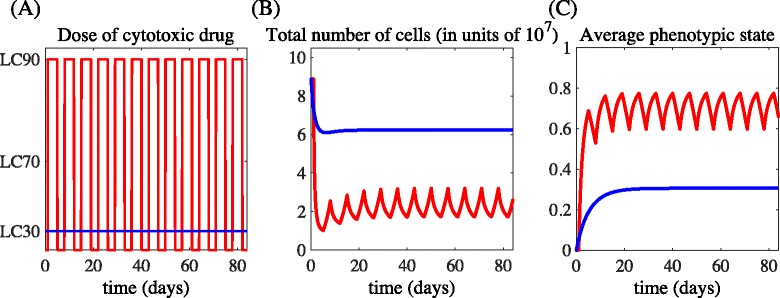
Comparing the administration of high-drug doses separated by drug-free periods with the continuous delivery of relatively low-drug doses. Sample dynamics of the total number of cells *ρ*(*t*) (panel **b**) and the average phenotypic state *μ*(*t*) (panel **c**) for the schedules of drug administration presented in panel (**a**). The red lines refer to the delivery of the drug dose equivalent to the LC_90_ for 4 days beginning on days 1, 8, 15, 22, 29, 36, 43, 50, 57, 64, 71 and 80, whereas the blue lines refer to the constant delivery of the drug dose equivalent to the the LC_30_. The values of the model parameters are as in Table 1, and the initial condition corresponds to the equilibrium population density for *c*(·)=0 [*i.e.*, the population density $\overline {n}(x)$ of Eq. (11)]

The results established by Theorem 2 demonstrate that higher doses of cytotoxic drug reduce the size of the cancer cell population at the cost of promoting the selection of more resistant phenotypic variants. Therefore, a natural question arises: how do therapeutic protocols relying on periodic phases of high-dose delivery separated by drug-free periods compare to protocols based on the administration of a continuous and relatively low drug dose? The results presented in Fig. 8 suggest that the first type of protocol causes a drastic reduction in the number of cancer cells, although it fosters an increase in the average level of resistance. On the other hand, the second type of protocol maintains a larger stable population of less resistant cells that suppress the growth of highly resistant clones. A more accurate comparison between the efficacy of the two therapeutic approaches would require a careful investigation of the respective adverse effects on healthy cells, which cannot be performed using our model. However, we can envisage a lower drug-induced toxicity being associated to the second type of protocols [50]. In the light of this observation, the analytical results of Theorem 2 and the numerical results summarised in Fig. 8 suggest that therapeutic protocols consisting of phases of high-dose delivery separated by drug-free periods may not offer the best long-term strategy for tumour control, and that protocols based on the continuous delivery of relatively low-drug doses may represent a suitable alternative.

## Conclusions

Although technological progress in molecular cell biology has resulted in large amounts of data documenting cancer progression, our understanding of the principles that underpin the development of resistance to anti-cancer agents and the emergence of phenotypic heterogeneity in neoplastic cell populations is filled with gaps and unresolved questions.

This work is presented with the perspective that mathematical modelling can help to address some of these gaps in our knowledge by capturing, in abstract terms, the crucial assumptions that underlie given hypotheses, and by offering alternative means of understanding experimental results that are currently available. With this perspective, our goal here is to contribute to a systematic identification of the way in which the phenotypic distribution, the level of intrapopulation heterogeneity and the size of cancer cell populations depend on the rate of random epimutations, the strength of natural selection, the intensity of the competition for resources, and the stress exerted by cytotoxic agents.

We have presented and analysed a PDE model of adaptive dynamics in a cancer cell population that is structured by the level of expression of a gene which is linked to cytotoxic-drug resistance and proliferative potential. While the parameter values that we have used to perform numerical simulations are derived from leukemia datasets, our analytical results are characterised by broad structural stability under parameter changes, and can accommodate parameter values from any other type of cancer.

The results of our analyses show that more intense non-genetic instability, stronger selection pressures and fierce competition for limited resources bring about cancer cell populations of smaller size. A more subtle question is how phenotypic heterogeneity is maintained or suppressed in cancer cell populations. Our model predicts that higher rates of random epimutations lead to higher phenotypic diversity, whilst intense selection pressures and high doses of cytotoxic drugs can deplete variation in the phenotypic pool of cancer cell populations, thus causing lower levels of intrapopulation heterogeneity. These findings recapitulate the results presented in previous theoretical and experimental studies of phenotypic evolution in neoplastic cell populations exposed to cytotoxic drugs [51–56].

Epigenetic mechanisms are increasingly implicated in the adaptation of neoplastic cells which face environmental changes. However, since epimutation rates are small in comparison to selection gradients, it might be speculated that the direction of evolution in cancer cell populations is determined exclusively by natural selection. On the contrary, our analytical results suggest that, in the presence of high concentrations of cytotoxic agents, frequent fluctuations in the cellular protein levels can trigger a type of ‘epimutational meltdown’ and contribute to extinction in non-fluctuating environments. This effect escalates under harsher environmental conditions (*i.e.*, for lower maximum fitness and higher selection gradients). Since we have derived a simple and general condition that can lead to population extinction – which is embodied by an explicit relation between the proliferation and death rates of cancer cells, the rate of epimutations and the concentration of the cytotoxic drug [*vid.*, point (i) of Theorem 2] – the validity of this argument can be tested in vitro. Because of the striking similarities between populations of cancer cells and microbial populations [57], an experimental setting analogous to that presented in [58] – where the authors made use of isogenic populations of engineered bacteria that randomly transitions between different phenotypic states to study adaptation to artificial environments – may prove to be useful for this purpose.

Here we have found that the same cell population exposed to different concentrations of the same cytotoxic drug can take different evolutionary trajectories, which culminate in the selection of phenotypic variants characterised by different levels of drug tolerance. This supports the notion that the response of cancer cells to cytotoxic agents is more complex than a simple binary outcome, *i.e.*, extinction of sensitive cells and selection of highly resistant cells, and agrees with insights from empirical works that highlight how different phenotypic variants can be selected inside the same solid tumour depending on the local concentrations of cytotoxic agents [59–61]. Also, in agreement with the experimental results presented by Sun et al. [62], our model suggests that cancer cell populations may regain sensitivity to cytotoxic drugs after a ‘drug holiday’.

In current clinical practice, it is common to give the maximum tolerated dose (MTD) of cytotoxic agents at the beginning of a therapy cycle, and then let the patient recover from toxicities. In the last decade, experimental and theoretical efforts have been made to determine the best scheduling of cytotoxic agents in anti-cancer therapy; unfortunately, there is still no clear answer about optimal administration protocols. Our work demonstrates that higher doses of cytotoxic drugs reduce the size of cancer cell populations at the cost of promoting the selection of more resistant phenotypic variants. Moreover, our analyses support the development of therapeutic approaches that go beyond the ‘MTD paradigm’. For instance, our results provide a theoretical basis for the benefits of metronomic therapy [63–66] – which relies on the continuous delivery of cytotoxic agents at relatively low, minimally toxic doses – and adaptive therapy [67] – which aims at enforcing a stable tumour burden by permitting sensitive cells to suppress highly resistant cells in low drug pressure conditions – as they may be more effective than traditional protocols at keeping the size of cancer cell populations under control while avoiding the expansion of drug tolerant clones.

We conclude with an outlook on possible extensions of the present work. Here, we have assumed that cell proliferation and drug resistance are interconnected. A natural way to extend our study would be to take into account a degree of independence in these processes by incorporating a second phenotypic variable. In this setting, we could also introduce an advection term to explore the effects of environmental stresses (*e.g.*, hypoxia, acidity or starvation) that may induce cells to actively acquire a more stress resistant phenotype. In so doing, we would be in the position to analyse how the interplay between selection, non-genetic instability and stress-induced adaptation governs the emergence of intra-tumour heterogeneity and the development of drug tolerance inside solid tumours, which is a theme that we began exploring in a previous paper [37].

The present model could also be extended to include the effects of the phenotypic coevolution and spatial interaction between cancer cells and cells of the tumour microenvironment. In fact, the growth and death of neoplastic cells is determined not only by their phenotype, but also by the microenvironment they inhabit. In particular, accumulating in vitro and in vivo evidence suggests that the interplay between cancer cells and stromal cells can induce mutual phenotype modifications promoting tumour growth and the emergence of resistance to anti-cancer agents [68–71]. As a first step, we may incorporate an additional structuring variable that stands for the spatial position of cells, and we could include an additional evolution equation for the local density of stromal cells, in order to investigate, *in silico*, the existence of ecological mechanisms which can foster the spreading of more proliferative and drug resistant phenotypic variants at the interface between solid tumours and host tissues.

Another track to follow to further enrich our model would be to include metabolic determinants of epigenetic dynamics in single cells. Simple phenomenological models have been proposed to describe the influence of oxygen and proton concentration in cancer tissues (see, for instance, [72, 73]). More recently the influence of metabolism on key epigenetic enzymes determining stemness (such as TET1/2, IDH1/2, DNMT3A) has been investigated in detail, setting the focus on DNA methylation dynamics [41, 74]. Mutations of such enzymes have been evidenced in leukemia and preleukemic states [75, 76]. Nevertheless, even without the occurrence of mutations, unequal repartition of these enzymes could be found to be a source of heterogeneity in cancer cell populations making cells diversely sensitive to their metabolic environment. Some cells, heavily loaded in these enzymes, may revert to a stem-like state, yielding germs for drug-resistant clones, while the others, not endowed with such abilities, would readily die when exposed to high doses of cytotoxic drugs. In this regard, mathematical modelling is still dependent on more biological knowledge to come about metabolism, stemness and epigenetic dynamics.

## Appendix 1. Proofs of Theorem 1 and Theorem 2

When *c*(·)=0, plugging definitions (6)–(7) into Eq. (3) we obtain

19 $$ \frac{\partial n}{\partial t} (x,t) = \beta \; \frac{\partial^{2} n}{\partial x^{2}} (x,t) + \left[\gamma - \eta \; x^{2} - d \; \rho(t)\right] \; n(x,t).  $$

The proof of Theorem 1 follows from a more general analysis that we have developed in two recent papers [33, 42], and it uses the results established by the following two lemmas:

### **Lemma 1**

If *γ*>(*βη*)^1/2^, the problem defined by completing (19) with (4)–(5) admits a unique non-negative nontrivial equilibrium solution $\overline {n}(x)$ which is given by (11).

### *Proof of Lemma 1*

Consider the PDE problem

20 $$ \left\{ \begin{array}{l} {\beta \; \overline{n}^{\prime\prime}(x) + \left[\gamma - \eta \; x^{2} - d \; \overline{\rho}\right] \; \overline{n}(x) = 0, \qquad x \in \mathbb{R},} \\ {\overline{\rho} = \int_{\mathbb{R}} \overline{n}(x) \; dx.} \end{array}\right.  $$

Writing

$$\overline{n}(x) = Y(z),\qquad z=\left(\frac{4\eta}{\beta}\right)^{1/4}x, $$

we find that *Y*(*z*) satisfies the differential equation

21 $$ Y^{\prime\prime}(z)-\left(\frac{z^{2}}{4}+a\right)Y(z)=0,  $$

with

$$a = \frac{d}{2(\beta\eta)^{1/2}}\left(\overline{\rho} - \frac{\gamma}{d}\right). $$

It is known that Eq. (21) has solutions which are bounded for all *z* if and only if *a*=−*m*−1/2, where *m* is a non-negative integer [77, 78]. These bounded solutions are the Gaussians exp(−*z*^2^/4) multiplied by polynomials of degree *m*, which form an orthogonal set of functions, and so are everywhere non-negative if and only if *m*=0. Therefore, the existence of a nontrivial non-negative solution of the PDE problem (20) requires *a*=−1/2. This implies that to have a nontrivial non-negative equilibrium solution the condition

$$\overline{\rho} = \frac{1}{d}\left[ \gamma - (\beta\eta)^{1/2} \right] $$

must be satisfied. If this condition is met, for some $A \in \mathbb {R}_{+}$

22 $$ \overline{n}(x) = A \exp \left\{-\frac{1}{2} \left(\frac{\eta}{\beta}\right)^{1/2}x^{2}\right\}.  $$

The constant *A* can be evaluated in terms of $\overline {\rho }$ by integrating Eq. (22). We find that

$$\overline{n}(x) = \overline{\rho} \; \frac{(\eta/\beta)^{1/4}}{(2\pi)^{1/2}}\exp\left\{-\frac{1}{2}\left(\frac{\eta}{\beta}\right)^{1/2} x^{2} \right\}, $$

and this concludes the proof of Lemma 1. □

### **Lemma 2**

The integral *ρ*(*t*) of the solution of the problem defined by completing (19) with (4)–(5) has the following long-time behaviour:

23 $$ {\lim}_{t \rightarrow \infty}\rho (t)= \left\{ \begin{array}{ll} {\frac{1}{d}\left[\gamma - (\beta\eta)^{1/2} \right]} & \text{if}\quad\gamma>(\eta\beta)^{1/2},\\ 0 & \text{if}\quad\gamma\leq (\eta\beta)^{1/2}. \end{array}\right.  $$

### *Proof of Lemma 2*

Following the method of proof that we presented in [33], it is possible to prove that, for all non-negative initial conditions *n*(*x*,0) such that 0<*ρ*(0)<*∞*,

$$\rho (t)=\frac{g(t)\rho(0)}{g(0)+ d \; \rho(0){\int_{0}^{t}} g(\tau) \; d\tau}, $$

with the function *g*(*t*) satisfying

$${} \begin{aligned} g(t)\sim \frac{2\beta}{(2\pi)^{1/2}} \int_{0}^{\infty} &\exp\left[-\frac{z^{2}}{4}\left(\frac{4\eta}{\beta}\right)^{1/4}z\right] \;dz \\ &\exp\left\{ \left[ \gamma - (\beta\eta)^{1/2} \right]t\right\}, \quad \text{as }\, t \rightarrow \infty. \end{aligned} $$

In the limit *t*→*∞*:

- if *γ*<(*βη*)^1/2^, then *g*(*t*)→0 exponentially rapidly;
- if *γ*=(*βη*)^1/2^, then *g*(*t*) converges to a positive constant;
- if *γ*>(*βη*)^1/2^, then *g*(*t*)→*∞* exponentially rapidly.

Therefore:

- if *γ*<(*βη*)^1/2^, then ${\lim \limits _{t \rightarrow \infty } \rho (t) = 0}$;
- if *γ*=(*βη*)^1/2^, then ${\lim \limits _{t \rightarrow \infty } \rho (t) = 0}$;
- if *γ*>(*βη*)^1/2^, then ${\lim \limits _{t \rightarrow \infty } \rho (t) = \frac {1}{d}\left [ \gamma - (\beta \eta)^{1/2} \right ]}$.

This concludes the proof of Lemma 2. □

Taken together, Lemma 1 and Lemma 2 allow us to reach the following conclusions:

(i) if *c*(·)=0 and *γ*−(*βη*)^1/2^≤0, then
  24 $$ \rho(t) \rightarrow 0, \qquad \text{as }\, t \rightarrow \infty;  $$
(ii) if *c*(·)=0 and *γ*−(*βη*)^1/2^>0, then
  $$\rho(t) \rightarrow \overline{\rho} = \frac{1}{d}\left[ \gamma-(\beta\eta)^{1/2} \right], \qquad \text{as }\, t \rightarrow \infty $$
  and
  $$\overline{n}(x) = \overline{\rho} \; \frac{{(\eta/\beta)^{1/4}}}{(2\pi)^{1/2}} \exp\left[-\frac{1}{2}\left(\frac{\eta}{\beta}\right)^{1/2} x^{2}\right]. $$

This establishes the claims of Theorem 1.

### Appendix 2: Proof of Theorem 2

When *c*(·)=*C*>0, plugging definitions (6)–(7) into Eq. (3) we obtain

$${} \begin{aligned} \frac{\partial n}{\partial t} (x,t) &= \beta \; \frac{\partial^{2} n}{\partial x^{2}} (x,t)\\ &\quad + \left[\gamma - \eta \; x^{2} - d \; \rho(t) - C \; (x-1)^{2}\right] \; n(x,t). \end{aligned} $$

Defining

$$\gamma_{c} := \gamma - \frac{\eta \; C}{\eta + C}, \qquad \eta_{c} := \eta + C \quad \text{and} \quad \overline{x}_{c} := \frac{C}{\eta+C}, $$

we can rewrite the above equation as

25 $$ \begin{aligned} \frac{\partial n}{\partial t}(x,t) &= \beta \; \frac{\partial^{2} n}{\partial x^{2}} (x,t)\\ &\quad + \left[\gamma_{c} - \eta_{c} \; (x-\overline{x}_{c})^{2} - d \; \rho(t)\right] \; n(x,t). \end{aligned}  $$

Since $x \in \mathbb {R}$, there is no loss of generality in translating coordinates so that $\overline {x}_{c}=0$. Hence, to adapt the method of proof of Theorem 1 to prove Theorem 2 is purely technical. For this reason, we leave Theorem 2 without proof.

## Reviewers’ comments

We thank the Reviewers for their comments, all of which are addressed below. Quotations from the Reviewers’ reports are italicised. It was apparent from the comments that some matters required clearer explanation, additional results or greater emphasis, so we have amended the manuscript appropriately.

### Reviewer 1 - Angela Pisco

In the present paper Lorenzi and co-authors offer a sound theoretical framework to address a critical point on development of drug resistance following treatment. The authors are able to numerically replicate the problem of cell state switching within cancer cell populations composed of cells with different sensitivities to drug. It is now becoming established that cancer treatment is a double edge sword, while the majority of cells die following chemo/radiotherapy, the surviving cells are no innocent bystanders and lead to a more aggressive tumour, mostly composed of cells with a stem-cell like phenotype that are multi-drug resistant. The authors’ original PDE model is able to replicate published experimental results taking into consideration both non-genetic and environmental conditions. Lorenzi et al. model simulations lead to import conclusions, namely the mathematical demonstration that higher drug doses promote resistant phenotypes and less heterogeneity within cancer cell populations. The entire work is novel and valuable for the community and because of that I’d endorse this paper for publication with minor changes. While I agree that the presented model is general enough for the conclusions to be valid in a panoply of cancers other than leukaemia, I’d like to inquire whether it is feasible with this model to replicate the cycle of sensitivity - resistance - sensitivity-... in line with what was described by Sun et al. [Reversible and adaptive resistance to BRAF(V600E) inhibition in melanoma, Nature, 2014].

Authors’ response: *We thank the Reviewer for this question. To address the Reviewer’s question, we have performed new numerical simulations considering piecewise-continuous periodic delivery of the cytotoxic drug. The results obtained are summarised by the movie ‘Additional file *2’* and are discussed at point R9 in the revised manuscript. In agreement with the experimental results presented by Sun et al., these results clearly show that administering the cytotoxic drug in an alternate fashion leads to the emergence of cycling subpopulations of drug-resistant and drug-sensitive cells.*

*All minor issues pointed out by the Reviewer have been addressed.*

### Reviewer 2 - Sébastien Benzekry

In their manuscript, Lorenzi et al. addressed, from a theoretical perspective, the fundamental issue of drug resistance in cancer cell populations. The article is well written and based on solid biological literature. The authors derived, mathematically analysed and simulated a phenotype-structured PDE model that essentializes key aspects of the problem, including the balance between level of resistance to the drug assault and proliferative rate. This is set in the context of adaptive dynamics driven by inheritable epigenetic changes. In doing so, they add another study to several works of them using the same methodology, with sometimes minor differences among them. Here, one interesting result obtained by the authors is that, depending on the dose of a cytotoxic drug delivered to the tumour, the average phenotypical trait toward which the population concentrates (a fact that is rigorously proven) is not always the same. This result is nontrivial and relevant since it demonstrates from basic and minimal biological hypotheses that the dosing has an impact on the phenotypic distribution of a cancer cell population, a topic with increasingly recognised importance for the treatment of cancers. However, no directly applicable recommendation can be derived for the clinical use of cytotoxic agents. I am not a biologist but I find it debatable that the cells closest to stemness (with *x*=1) are the ones having the smallest proliferative ability. From my understanding, stem cells could be at the same time more resistant and more proliferatively active.

Authors’ response: *We thank the Reviewer for having stressed this point. Following the advice of Reviewer 3, we do not mention stemness anymore in the revised manuscript, as it is unnecessary for our conclusions to hold. Therefore, we state that the phenotypic state x=1 corresponds to the highest level of cytotoxic-drug resistance, without making any reference to stemness.*

I don’t understand why functions *k* and *p* have to be respectively convex and concave. Wouldn’t it be enough to consider them decreasing/increasing (as functions of the norm of *x*)?

Authors’ response: *This may be an alternative modelling strategy and, from the mathematical perspective, it may be interesting to explore it. However, concavity and convexity assumptions lead naturally to smooth fitness landscapes which are close to the approximate fitness landscapes inferred from experimental data through regression techniques [see, for instance, Otwinowski, J., and Plotkin, J.B. (2014). Inferring fitness landscapes by regression produces biased estimates of epistasis. Proc. Natl. Acad. Sci. USA 111(22), E2301–E2309 and references therein].*

I also find it surprising, if not inconsistent, that the proliferation rate p(x) can take negative values, while being defined to be the “proliferation due to the assumed velocity of the cell cycle in the absence of between-cell competition for nutrients, and in the absence of drug-induced death”. This appears to me to be the underlying reason of the possibility of extinction of the population (first part of Theorem 1), which is quite surprising and sounds biologically unrealistic, in the absence of treatment.

Authors’ response: *We thank the Reviewer for his observation. As stated both in the old version version of the manuscript and in the updated manuscript, the function p*(*x) “stands for the net proliferation rate of cancer cells in the phenotypic state x”. The sentence quoted by the Reviewer from the old version of manuscript was not pertinent and it has been removed from the updated manuscript.*

In Table 1, units of the parameters should be provided, when appropriate. What motivated the choice of *K*=10^8^ cells (no reference is provided)?

Authors’ response: *Units have been included in Table 1, and a reference motivating the choice of the value of the carrying capacity K has been added.*

What biological situation is modelled here: an in vitro cell line? in vivo tumours in animal models? a clinical patient tumour?

Authors’ response: *We thank the Reviewer for this observation. We have clarified this point. As stated in the updated manuscript, we consider an in vitro culture system.*

I appreciate the efforts of the authors to adopt relevant values of the parameters based on the literature. However, the value of *γ* taken seems particularly high (it gives a doubling time of *log*(2)/*gamma*∗24=3.34 hours, which is much faster than the fastest mammalian cell cycle time [1]). This explains why, in Fig. 1b, the cell population reaches the carrying capacity so fast, probably much faster than in the biological reality. The authors might want to choose a more meaningful value for the duration of the fittest cancer cells’ cycle duration, such as about 25 h. [1] Steel, G. G. and Lamerton, L. F. (1966). The growth rate of human tumours. Br. J. Cancer, 20(1):74–86

Authors’ response: *We thank the Reviewer for this suggestion. Following the Reviewer’s advice, we updated the value of the parameter γ so that the duration of the fittest cancer cells’ cycle duration is about 25 h. Because of this, we updated also the values of the parametersβ andη following the reasonings presented in the updated manuscript, and we rerun all simulations. Therefore, all figures in the paper have been updated.*

*All minor issues pointed out by the Reviewers have been addressed.*

### Reviewer 3 - Heiko Enderling

Lorenzi et al. present a mathematical model of phenotypically structured cancer cell populations, and track the evolution of phenotype heterogeneity in the absence of or under selection pressure of a cytotoxic drug. The model is based on previous literature and extended to study continuous heterogeneity and plasticity. The manuscript is well prepared, the work is coherently presented, and the results are clearly discussed. Most of the results are as one would expect, attributing to careful model development. Some results are intriguing, for example indications that maximum tolerable dose may not be the best long-term strategy for tumour control, and that a metronomic scheme may provide a suitable alternative. While certainly plausible, these scenarios can be readily explored with the model and should be included in the manuscript. In particular, it would be interesting to see the temporal evolution of trade-offs between tumour volume reduction and selection for resistance in an MTD vs. metronomic setting.

Authors’ response: *We thank the Reviewer for this suggestion. To address the Reviewer’s question, we have performed new numerical simulations comparing the administration of high-drug doses separated by drug-free periods with the continuous delivery of relatively low-drug doses. The results obtained are summarised by Fig. *8* and are discussed at point R10 in the revised manuscript. These results suggest that the first type of protocol causes a drastic reduction in the number of cancer cells, although it fosters an increase in the average level of resistance in the population. On the other hand, the second type of protocol maintains a larger tumour burden characterised by a relatively lower average level of resistance. A more accurate comparison between the efficacy of the two therapeutic approaches would require a careful investigation of the respective adverse effects on healthy cells, which cannot be performed using our model. However, we can envisage a lower drug-induced toxicity being associated to the second type of protocols [see, for instance, Gately, S., Kerbel, R. (2001). Antiangiogenic scheduling of lower dose cancer chemotherapy. Cancer J., 7(5), 427–436]. In the light of this observation, the analytical results of Theorem 2 and the numerical results summarised in Fig. *8* suggest that therapeutic protocols consisting of phases of high-dose delivery separated by drug-free periods may not offer the best long-term strategy for tumour control, and that protocols based on the continuous delivery of relatively low-drug doses may represent a suitable alternative.*

My major critique is the discussion of cancer stem cells for treatment resistance. The authors introduce a trade off between proliferation and quiescence opposing a gradient of differentiated and cancer stem cells. While this might be biologically realistic, the developed mathematical model does not reflect a cellular hierarchy. In contrast, in the model as is, a population of non-stem cancer cells would grow infinitely in the absence of an initiating and propagating cancer stem cell – a clear violation of the biological concept. As the presented model, results and discussion are solely dependent and reproducible with a proliferation gradient alone, I recommend the stem cell component to be dropped. The often-attributed cancer stem cell quiescence may be discussed as further support for applicability of the model at the end of the manuscript.

Authors’ response: *We thank the Reviewer for this observation. Following the Reviewer’s advice, we do not make any reference to stemness in the revised manuscript, as it is unnecessary for our conclusions to hold.*

## Additional files

Additional file 1 Evolution of an initial population which is mainly composed of cells in the phenotypic state that corresponds to the highest level of cytotoxic-drug resistance, in the absence of xenobiotic agents. This movie shows the evolution of an initial population which is mainly composed of cells in the phenotypic state *x*=1 (*i.e.*, the phenotypic state that corresponds to the highest level of cytotoxic-drug resistance) for *c*(·)=0 (*i.e.*, in the absence of xenobiotic agents). The values of the model parameters are as in Table 1. The pink line indicates the equilibrium population density for *c*(·)=0 [*i.e.*, the population density $\overline {n}(x)$ of Eq. (11)]. In agreement with the experimental results presented in [14], it takes approximatively 18 days for the population to reconstitute the equilibrium phenotypic distribution. (MOV 203 kb)

Additional file 2 The cancer cell population regains cytotoxic-drug sensitivity when drug delivery is discontinued. This movie shows the evolution of an initial population which is mainly composed of highly proliferative cells (*i.e.*, cells in the phenotypic state *x*=0) exposed to periodic phases of high-dose delivery (drug dose corresponding to the LC_90_) separated by drug-free periods. The values of the model parameters are as in Table 1. The red and blue lines highlight the highly proliferative state *x*=0 and the highly drug-resistant state *x*=1. In agreement with the experimental results presented in [62], these results clearly show that administering the cytotoxic drug in an alternate fashion leads to the emergence of cycling subpopulations of drug-resistant and drug-sensitive cells. (MOV 415 kb)
